# Allelic Variation in the Toll-Like Receptor Adaptor Protein *Ticam2* Contributes to SARS-Coronavirus Pathogenesis in Mice

**DOI:** 10.1534/g3.117.041434

**Published:** 2017-06-05

**Authors:** Lisa E. Gralinski, Vineet D. Menachery, Andrew P. Morgan, Allison L. Totura, Anne Beall, Jacob Kocher, Jessica Plante, D. Corinne Harrison-Shostak, Alexandra Schäfer, Fernando Pardo-Manuel de Villena, Martin T. Ferris, Ralph S. Baric

**Affiliations:** * Department of Epidemiology, University of North Carolina, Chapel Hill, North Carolina 27599; † Department of Genetics, University of North Carolina, Chapel Hill, North Carolina 27599; ‡ Department of Microbiology and Immunology, University of North Carolina, Chapel Hill, North Carolina 27599; § Lineberger Comprehensive Cancer Center, University of North Carolina, Chapel Hill, North Carolina 27599

**Keywords:** SARS-CoV, Collaborative Cross, F2, Ticam2, host susceptibility genes, Multi-parent Advanced Generation Inter-Cross (MAGIC), multiparental populations, MPP

## Abstract

Host genetic variation is known to contribute to differential pathogenesis following infection. Mouse models allow direct assessment of host genetic factors responsible for susceptibility to Severe Acute Respiratory Syndrome coronavirus (SARS-CoV). Based on an assessment of early stage lines from the Collaborative Cross mouse multi-parent population, we identified two lines showing highly divergent susceptibilities to SARS-CoV: the resistant CC003/Unc and the susceptible CC053/Unc. We generated 264 F2 mice between these strains, and infected them with SARS-CoV. Weight loss, pulmonary hemorrhage, and viral load were all highly correlated disease phenotypes. We identified a quantitative trait locus of major effect on chromosome 18 (27.1–58.6 Mb) which affected weight loss, viral titer and hemorrhage. Additionally, each of these three phenotypes had distinct quantitative trait loci [Chr 9 (weight loss), Chrs 7 and 12 (virus titer), and Chr 15 (hemorrhage)]. We identified *Ticam2*, an adaptor protein in the TLR signaling pathways, as a candidate driving differential disease at the Chr 18 locus. *Ticam2*^−/−^ mice were highly susceptible to SARS-CoV infection, exhibiting increased weight loss and more pulmonary hemorrhage than control mice. These results indicate a critical role for *Ticam2* in SARS-CoV disease, and highlight the importance of host genetic variation in disease responses.

Severe Acute Respiratory Syndrome-Coronavirus (SARS-CoV) emerged in 2002–2003 as the first highly pathogenic zoonotic virus of the 21st century (Ksiazek *et al.* 2003). During the outbreak, over 8000 people were infected with SARS-CoV (https://www.cdc.gov/sars/about/fs-sars.html), and these individuals experienced disease phenotypes ranging from mild respiratory symptoms to severe pulmonary disease including diffuse alveolar damage (DAD), acute respiratory distress syndrome (ARDS), and death (10% mortality rate). More recently, a close relative to SARS-CoV, designated Middle East Respiratory Syndrome-CoV (MERS-CoV) was identified from a patient hospitalized with pneumonia in Saudi Arabia (Zaki *et al.* 2012). Since 2012, >1850 MERS cases (http://www.who.int/emergencies/mers-cov/en/) have been identified, with sporadic cases still appearing in the Middle East. MERS-CoV infection has a 35% mortality rate, making it the second highly pathogenic human coronavirus, and supporting the hypothesis that emerging coronaviruses threaten global health. Additionally, a large number of SARS-like coronaviruses have been identified in geographically diverse bat populations, highlighting the potential for related pathogens to emerge into the human population (Ge *et al.* 2013; Menachery *et al.* 2015; Yang *et al.* 2015; Kim *et al.* 2016; Menachery *et al.* 2016). While SARS-CoV and MERS-CoV both cause significant morbidity and mortality, the limited scope of the outbreaks greatly restricted our understanding of the host and viral factors that contribute to coronavirus-induced disease.

Given the sporadic nature of emerging disease outbreaks, new strategies are needed to identify host genetic variants that regulate disease severity and susceptibility. Host genetic variation contributes to susceptibility to many infectious diseases in human populations (Chapman and Hill 2012; Manry and Quintana-Murci 2013), including SARS-CoV (Ip *et al.* 2005; Zhang *et al.* 2005; Chen *et al.* 2006). However, inability to control for important confounding factors—including dose, route of infection, various comorbidities (age, weight, etc.), and baseline immune state of the host—complicate interpretation of genome-wide association studies in human populations. While these complex factors are often unknown in human infections, small animal models of disease allow for controlled experiments to dissect the influence of such variables, and have greatly expanded our understanding of SARS-CoV pathogenesis (Roberts *et al.* 2005a,b, 2007). Candidate gene approaches and careful analysis of the host immune response to infection (Frieman *et al.* 2007; Sheahan *et al.* 2008; Zhao *et al.* 2011; Totura *et al.* 2015) have revealed the importance of a number of classical aspects of both the innate and adaptive immune response in facilitating aspects of SARS-CoV protection and disease enhancement. In addition, predictive systems approaches have highlighted less obvious aspects, such as wound-repair, in influencing host outcomes following SARS-CoV infection (Gralinski *et al.* 2013).

Pattern recognition receptors (PRRs), such as toll-like receptors (TLRs), complement receptors, and RIG-I-like receptors trigger the innate immune response to respond to pathogens (Adachi *et al.* 1998; Poltorak *et al.* 1998; Alexopoulou *et al.* 2001; Akira *et al.* 2006; Kawai and Akira 2009; Dunkelberger and Song 2010). PRRs recognize foreign molecules in the cytoplasm, endosome, or at the cell surface specific to invading pathogens such as lipopolysaccharide (LPS), flagellin, and double stranded RNA. Not all identified PRRs have known ligands (Oosting *et al.* 2014), and new PRRs and ligands are still being discovered. SARS-CoV infection is sensed by TLR3 and TLR4 (Totura *et al.* 2015), and SARS-CoV actively evades detection of its cytosolic RNA by MDA5 through the 2′ methyltransferase activity of the viral nsp16 protein (Menachery *et al.* 2014). Furthermore, numerous SARS-CoV genes block interferon sensing and signaling (Kopecky-Bromberg *et al.* 2007; Frieman *et al.* 2009; Clementz *et al.* 2010) among other immune evasion strategies. Candidate gene studies of PRRs or other immune factors in inbred mouse strains typically use genetic knockouts, which are rare in natural populations, and do not reflect natural genetic variation. Incongruent disease phenotypes have also been observed when gene knockouts are analyzed on the background of different inbred strains (Bonyadi *et al.* 1997; Linder 2006; Barbaric *et al.* 2007; Ramsbottom *et al.* 2015), thus complicating the interpretation of those results to outbred populations like humans. Functional knockouts in human immune genes are unusual but do exist (Godowski *et al.* 1989; Greenberg *et al.* 1991; Conrad *et al.* 2006; Knight 2013). *CCR5Δ32* is a rare example of a nonfunctional gene being advantageous, leading to its spread throughout the population (Novembre *et al.* 2005). More commonly, such mutations are found to have deleterious effects, such as in the case of *TLR3* deficiency leading to susceptibility to Herpes Simplex Virus 1 - induced encephalitis (Zhang *et al.* 2007), *IRAK-4* deficiency resulting in susceptibility to bacterial infections (Picard *et al.* 2003), or the *CDK4* deletions found in cancer patients (Nobori *et al.* 1994). More relevant for human disease is the impact of functional allelic variation in both coding and noncoding regions of genes, which the Collaborative Cross (CC) is designed to model.

Inbred mouse strains have represented the gold standard in animal model development, designed primarily to minimize experimental variables in a mammalian system (Chung *et al.* 1997; Rosenthal and Brown 2007; Vandamme 2014). However, the limited genetic variation segregating among classical inbred strains such as C57BL/6 and Balb/c and their convoluted ancestry limits their use for genetic association studies (Williams *et al.* 2004; Yang *et al.* 2007, 2011). In recent years, there has been a growing appreciation for the importance of genetic variation between individuals in contributing to a number of disease states including autoimmune diseases, Alzheimer’s disease and general immunodeficiency (Rebeck *et al.* 1993; Schmalstieg and Goldman 2002; Lee *et al.* 2013). The CC is a multi-parent population (MPP) of recombinant inbred strains created to assess and identify genetic variants driving complex disease, while concurrently maintaining the reproducibility and manipulative potential of inbred strains (Threadgill *et al.* 2011; Threadgill and Churchill 2012b). Each CC strain is a unique mosaic of eight founder haplotypes—A/J, C57BL/6J, 129S1/SvImJ, NOD/ShiLtJ, NZO/HlLtJ, CAST/EiJ, PWK/PhJ and WSB/EiJ—representing all three subspecies of house mouse; >40 million genetic variants segregate in the CC population (Keane *et al.* 2011) (Srivastava *et al.* 2017) (Oreper *et al.* 2017). Recently, a wealth of information on the genetic architecture of immune responses and viral disease pathogenesis has been identified in populations related to the CC (Durrant *et al.* 2011; Ferris *et al.* 2013; Phillippi *et al.* 2014; Xiong *et al.* 2014; Graham *et al.* 2015; Gralinski *et al.* 2015; Graham *et al.* 2016)(Green *et al.* 2017). We previously identified numerous host genetic loci that contribute to SARS-CoV pathogenesis using a screen of the incipient lines of the CC (the preCC) (Gralinski *et al.* 2015).

Here, we extend our work from the preCC population to an F2 cross between two inbred CC strains showing extreme divergent responses to SARS-CoV (Gralinski *et al.* 2015), the first F2 study of infectious disease between two CC lines. In contrast to QTL mapping across a genetic reference population, focused F2 crosses allow for a more complete dissection of extremely divergent phenotypic responses between pairs of strains, an approach that can highlight multi-genic and complex interactions in a more focused (and powered) contrast. The phenotypic distribution for each SARS-CoV response trait we measured equaled or exceeded the distribution seen between the two parent strains of the F2, and the phenotypes were broad (over a four log range in viral titer levels and >30% difference in weight loss in response to infection). Quantitative trait loci (QTL) mapping identified five significant loci contributing to weight loss, virus titer, pulmonary hemorrhage, and histopathology phenotypes, and we found evidence for both additive and epistatic interactions between these loci. A QTL affecting multiple SARS-CoV response traits on chromosome 18 from 27.1–58.6 Mb that contributed between 6 and 12% of each phenotype was selected for further study. Bioinformatics analysis reduced the number of candidate genes in the QTL region, leading to prioritization of *Ticam2* for candidate gene studies. We confirmed that *Ticam2*, a TLR adapter protein specific to TLR4, contributes to SARS-CoV pathogenesis by showing that *Ticam2*^−/−^ mice have increased susceptibility to SARS-CoV infection. By using CC lines with extreme SARS-CoV response phenotypes, we may have enriched for extreme alleles selected from different pairs of founders at each causative locus. Our data reaffirms use of F2 crosses as a powerful strategy to identify novel genetic variants that regulate extreme disease phenotypes following virus infection in the CC resource population.

## Materials and Methods

### Virus and cells

Recombinant mouse-adapted SARS-CoV (MA15) was propagated on Vero E6 cells. For virus titration, the lower half of the right lung was homogenized in PBS and plated for plaque assay using Vero E6 cells to give plaque forming units (PFU) per lung with a detection limit of 100 PFU (Deming *et al.* 2006). All experiments were performed in a class II biological safety cabinet in a certified biosafety level 3 laboratory containing redundant exhaust fans by workers wearing personnel protective equipment, including Tyvek suits, hoods, and high-efficiency particulate air (HEPA)-filtered powered air-purifying respirators (PAPRs).

### Animals

PreCC mice were infected and assayed as described previously (Gralinski *et al.* 2015). CC003/Unc and CC053/Unc mice were obtained from the UNC Systems Genetics Core. F1 and F2 mice were bred in house from these two parent lines, and infected at 9–11 wk of age. Both male and female mice were used for F1 and F2 studies, while the preCC used only female mice. F2 mice were identified by earpunch and randomly cohoused at the time of weaning; a tail snip for DNA extraction was also taken at that time. *Ticam2*-deficient mice on a C57BL/6 background were obtained from the Heise laboratory (UNC), originally created by Yamamoto *et al.* (2003). All mice were anesthetized with a mixture of ketamine and xylazine, intranasally infected with 10^5^ PFU of MA15 in a 50 μl volume, and weighed daily. Mice were acclimated to BSL3 housing for a minimum of 7 d prior to infection. All mouse studies were performed at the University of North Carolina (Animal Welfare Assurance #A3410-01) using protocols approved by the UNC Institutional Animal Care and Use Committee (IACUC).

### Histological analysis and hemorrhage

Gross pulmonary hemorrhage was observed at the time of tissue harvest, and scored on a scale of 0 (no hemorrhage in any lobe) to 4 (extreme and complete hemorrhage in all lobes of the lung). Lung tissues for histological analysis were fixed in 10% formalin for at least 7 d, embedded in paraffin, and 5-µm sections were prepared by the UNC histopathology core facility. To determine the extent of inflammation, sections were stained with hematoxylin and eosin (H&E), and scored in a blinded manner as previously described (Gralinski *et al.* 2013).

#### DNA isolation and genotyping:

Genomic DNA was isolated from tail tissue using the Qiagen (Hilden, Germany) DNeasy Blood & Tissue kit protocol, and was quantified and assessed for purity using a Nanodrop instrument (Thermo-Fisher Scientific). Genomic DNA (∼1.5 μg) was sent from each animal to Neogen Inc. (Lincoln, NE) for array hybridization on the MUGA array (Morgan *et al.* 2015). Genotypes were called by the vendor using the GenCall algorithm implemented in the Illumina BeadStudio software. Quality checks and further analysis used the *argyle* package (Morgan 2015) for the R environment (www.cran.r-project.org).

#### QTL mapping:

We selected those SNP markers behaving in a biallelic manner between replicate samples of CC003/Unc and CC053/Unc (Threadgill and Churchill 2012a), and, using the *argyle* package, we used the thin.genotypes() function to arrive at a set of 304 biallelic markers evenly spaced across the genome for QTL mapping. We exported these data into the *R/QTL* (Arends *et al.* 2010) package using argyle’s *as.rqtl.genotypes()* function, and mapped QTL for each of the measured phenotypic traits using the scanone() function in *rqtl*. Specifically, the scanone() function fits a model:

$$y_{i}=m+Bx_{i}+\varepsilon$$

where *y_i_* is the phenotypic value of individual *i*, *m* is the population mean, *x_i_* is the genotype at a putative QTL, and *ε* is the error term, with *Β* being the estimated effect of transitioning from one allele to another at the putative QTL. Scanone() uses standard interval mapping (Lander and Botstein 1989) to assess the significance of fit of this model relative to the null model:

$$y_{i}=m+\varepsilon .$$

Significance was assessed for each phenotype using 500 permutations. QTL regions were denoted using a 1.5 LOD-drop method.

We next utilized the scantwo() function of R/QTL to assess the likelihood of higher order interactions between pairs of loci. Scantwo() fits a series of models looking at the fit of two loci as a full model:

$$y_{i}=m+B_{a}x_{1i}+B_{b}x_{2i}+B_{c}\left\{x_{1i}*x_{2i}\right\}+\varepsilon$$

where *x*_1_*_i_* and *x*_2_*_i_* are the genotypes at two putative loci, and *x*_1__i_*x_2_*_i_* is a representation of the combination of these two genotypes. In this case, *B*_a_ is the estimated effect of transitioning between alleles at putative locus 1, *B*_b_ the estimated effect of transitioning between alleles at putative locus 2, and *B*_c_ is the estimated interaction effect of transitioning between alleles within each locus. Scantwo() also assesses the fit of an additive model:

$$y_{i}=m+B_{a}x_{1i}+B_{b}x_{2i}+e$$

Both of these models, as well as a pure-interaction model (Full model fit-Additive model fit) are then assessed relative to a null model:

$$y_{i}=m+e.$$

Significance in these situations was assessed using a total of 250 permutations.

#### Statistical analysis:

SARS-CoV F2 phenotypes were compared by Pearson correlation using Graphpad Prism, and raw *P*-values are reported. *Ticam2*^−/−^ and C57BL/6J phenotypes were compared by unpaired Student’s *t*-test. The percentage of phenotypic variation each QTL contributed (as reported in Table 1) were assessed using the lm() function in R, and determining the SS_genotype_/SS_total_ fraction at each peak marker at a QTL.

**Table 1:** QTL regions and statistics

QTL	Trait(s)	Chromosome	Start (Mb)	Max (Mb) and Marker	Stop (Mb)	Percent Variation Explained (%)
*HrS5*	D3% weight	Chr 18	27.108062	42.852536 backupUNC181069094	58.694005	6.60
	D4% weight		27.108062	51.250937 JAX00083358	58.694005	8.50
	Log titer		27.108062	51.250937 JAX00083358	58.694005	12.90
	Hemorrhage		24.762824	51.250937 JAX00083358	78.29634	6
*HrS6*	D3% weight	Chr 9	116.476207	121.771517 backupJAX00708075	Telomere	7
*HrS7*	Log titer	Chr 7	55.169841	96.668697 UNC070369595	117.22358	12.30
*HrS8*	Log titer	Chr 12	81.649471	88.541688 UNC120199018	108.529109	5.40
*HrS9*	Hemorrhage	Chr 15	Centromere	30.785867 UNC150077326	64.430001	9.10

### Data availability

Complete F2 phenotypes, along with the subset of genotypic markers used for mapping, are available in Supplemental Material, Table S1. F1 and *Ticam2*^−/−^ data are available in Table S3. Genotype data are available at Zenodo (DOI 10.5281/zenodo.401060) and also at http://www.med.unc.edu/mmrrc/genotypes/. QTL outputs for D3 weight loss, D4 weight loss, Log_10_ Titer, and hemorrhage are available in Table S4, Table S5, Table S6, and Table S7.

## Results

**Figure 1:**
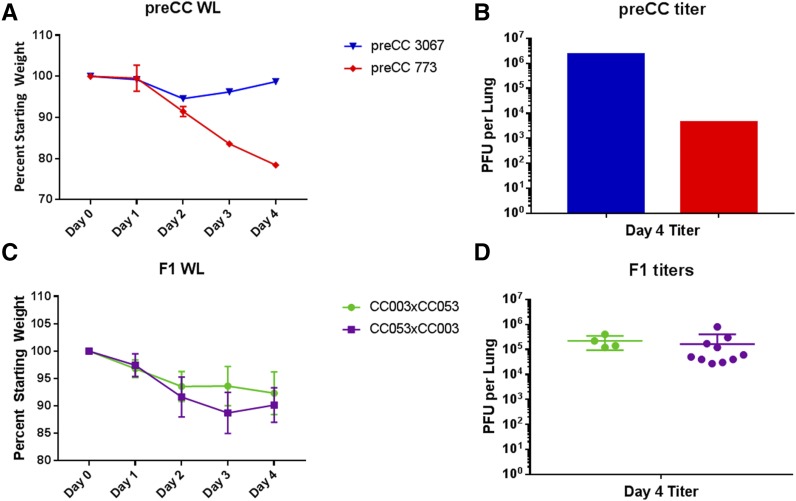
preCC parent and F1 phenotypes. preCC mice from lines 3067 (*n* = 1) and 773 (*n* = 2) were infected with 10^5^ PFU of MA15 and followed for overall pathogenesis as measured by weight loss relative to day zero (A) and virus titer in the lung at day four (B). Two animals from line 773 were received; however, one succumbed to infection at day 3 postinfection. Weight loss (C) and titer (D) were tested in reciprocal F1 mice [CC003×CC053 (*n* = 7 for WL and *n* = 4 for titer) and CC053×CC003 (*n* = 14 for WL and *n* = 12 for titer)].

We selected two CC strains (CC003/Unc and CC053/Unc) which (a) had shown extreme and divergent SARS-CoV responses in our preCC study (Gralinski *et al.* 2015), and (b) were available as completely inbred stains at the time we initiated this study [many preCC strains went extinct during the inbreeding process (Shorter *et al.* 2017)]. PreCC funnel 3067, now the fully inbred line CC003/Unc, was highly resistant to SARS-CoV-induced weight loss (Figure 1A), despite having a high viral load in the lung at 4 d postinfection (Figure 1B). In contrast, preCC funnel 773, now the fully inbred line CC053/Unc, was highly susceptible to SARS-CoV infection, exhibiting extreme weight loss and mortality, but a low virus load in the lung. From CC003/Unc and CC053/Unc, we bred reciprocal F1 mice to test for susceptibility to SARS-CoV infection. Both male and female F1 mice were intranasally infected with 10^5^ PFU of mouse-adapted SARS-CoV (MA15). All F1 animals showed intermediate weight loss and titer phenotypes (Figure 1, C and D and raw data in Table S2), and these phenotypes were highly similar regardless of the cross order. Together, the results suggested that genetic elements driving susceptibility and resistance in parental lines were not dominant, not dependent on parent of origin, and could be mapped in an F2 cross.

### Infected F2 progeny produce a range of disease

**Figure 2:**
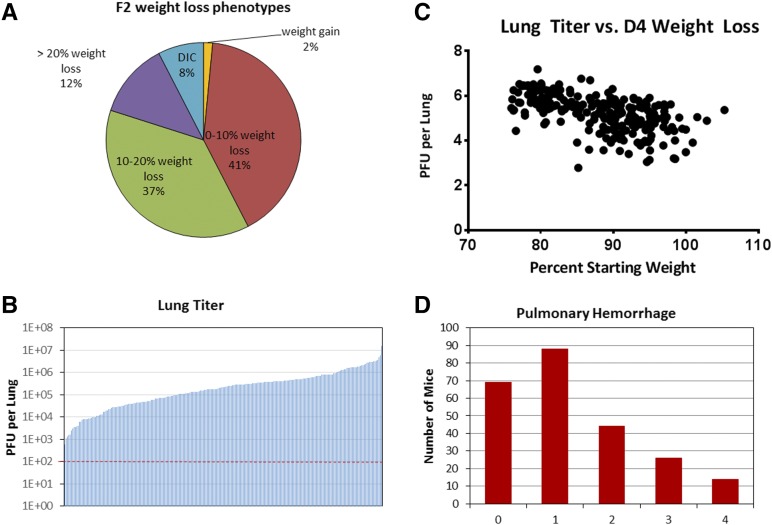
F2 phenotypes. F2 mice were infected with 10^5^ PFU of MA15 and monitored for 4 d. Percent starting weight as measured at day 4 is shown in (A) and virus titer is shown in (B). A significant correlation was observed between weight loss and titer as shown in (C). Pulmonary hemorrhage was scored at the time of harvest and is shown in (D).

F1 mice were bred to generate 264 F2 mice for challenge with SARS-CoV. All mice were infected with 10^5^ PFU of mouse-adapted SARS-CoV (MA15) at 9–11 wk of age, and monitored daily for weight loss and signs of disease until harvest at 4 d postinfection. Unlike the F1 mice, which had a narrow range of disease, F2 mice showed expanded phenotypes, exceeding both the range of weight loss and titer observed in the parents (Figure 2). Two percent (4/264) of F2 mice gained weight over the course of the 4 d infection, and an additional 41% (108/264) of F2 animals lost 0–10% of their starting weight, marking them as relatively resistant to infection (Figure 2A). Thirty-seven percent (99/264) of animals were moderately susceptible, losing between 10 and 20% of their starting weight by day 4 postinfection. Twenty percent of animals were extremely susceptible to infection, losing either over 20% of their starting weight (12%, 33/264) or succumbing to infection (8%, 20/264). Notably, 28% of F2 mice showed transient weight loss, and began to recover from infection by day 4; they are represented in both the 0–10% and 10–20% weight loss groups. Overall, the weight loss results highlight the range and diversity of the F2 progeny’s host response to SARS-CoV infection.

In addition to weight loss, other markers of pathogenesis demonstrate the variability of the F2 response to SARS-CoV. Lung titers were assessed by plaque assay for all surviving F2 mice, and titers ranged from <10^3^ PFU per lung to >10^7^ PFU per lung (Figure 2B). Notably, significant correlation was observed between weight loss and titer (Pearson’s *r* −0.5553, *P* < 0.0001, Figure 2C) at day 4 postinfection. Both male and female F2 mice had similar ranges of weight loss and virus load in the lung (Figure S1); in addition, both showed significant correlations between the two phenotypes. Pulmonary hemorrhage was assessed at the time of tissue harvest, and illustrated a spectrum of disease. F2 mice ranged from no hemorrhage (85 mice with a score of zero) (Figure 2D) to extreme hemorrhage (42 mice with a score of 3 or 4). Hemorrhage was significantly correlated with both day 4 weight loss (*r* of −0.699, *P* < 0.0001) and titer (*r* of 0.487, *P* < 0.0001). Table S1 contains the full phenotypic data for all F2 mice including lung histopathology scoring.

### Mapping F2 phenotypes reveals multiple QTL

**Figure 3:**
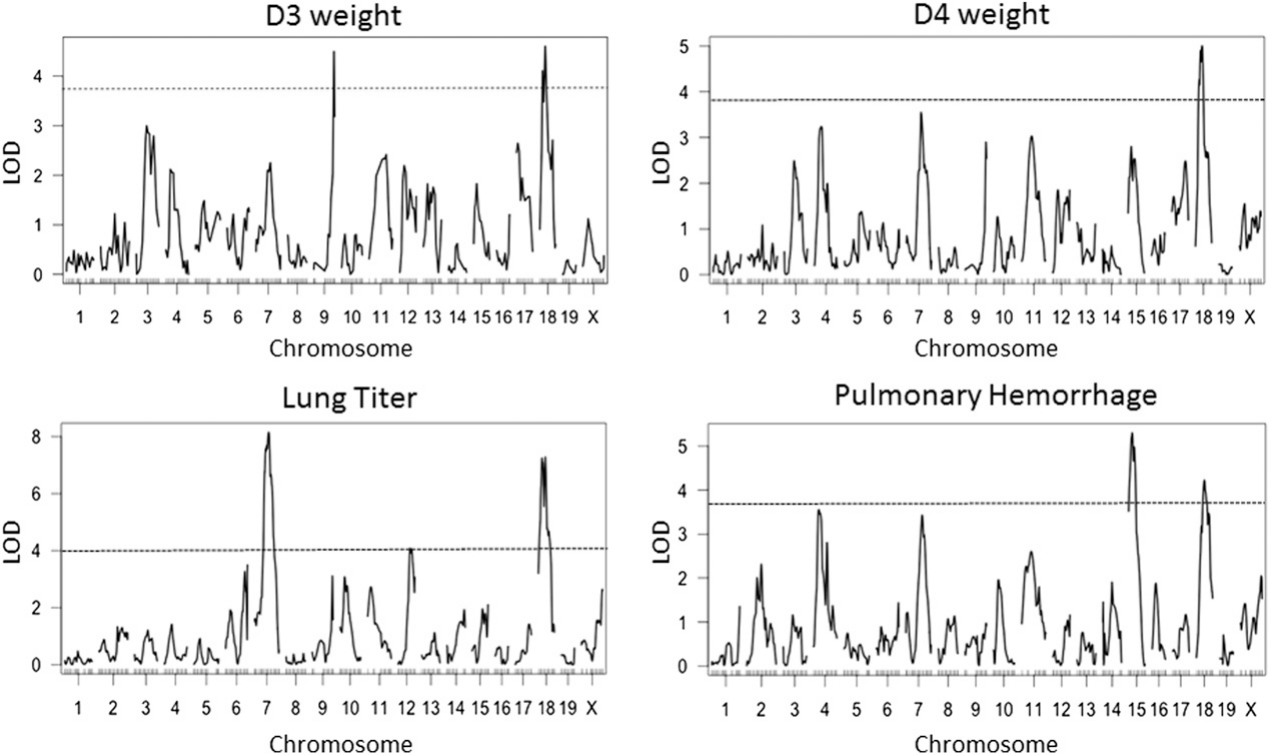
SARS QTL. QTL analysis using the F2 phenotypes and genotypes revealed multiple QTL. The dashed line indicates a significance value of 0.05 as determined by permutation test.

F2 mice were genotyped using the MUGA array, and we conducted QTL mapping using 304 evenly spaced and informative markers using R/QTL (Arends *et al.* 2010). We identified five QTL associated with a variety of traits: one locus on Chr*18* (Host response to SARS QTL #5, *Hrs5*) was associated with weight loss at day 3 and day 4 postinfection, viral titer, pulmonary hemorrhage (Figure 3), vascular cuffing, and edema histopathology phenotypes (Figure S2). Trait-specific QTL were also identified for day 3 weight loss (*Hrs6* Chr *9*), viral titer (*Hrs7* Chr *7*, *Hrs8* Chr *12*), and hemorrhage (*Hrs9* Chr *15*). Analysis of the chromosome *18* multi-trait QTL indicated a phenotypic contribution of 6.6% of day 3 weight loss, 8.5% of day 4 weight loss, 12.9% of variation in viral titer, 6% of hemorrhage, and a consensus region of 27.1–58.6 Mb (all QTL are summarized in Table 1). Table S4, Table S5, Table S6, and Table S7 show the complete QTL mapping files, with the LOD score at each of the 304 markers used.

**Figure 4:**
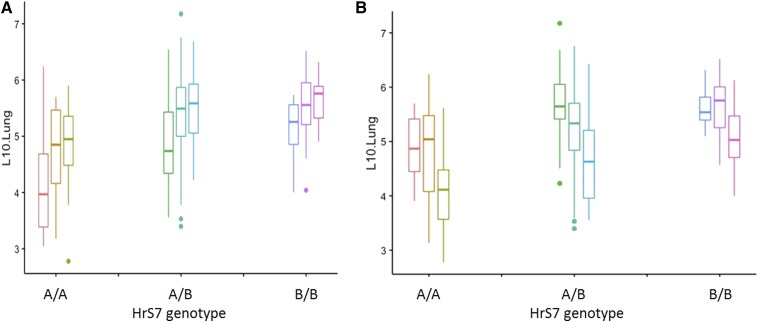
Interactions between loci driving viral titer responses. (A) Interactions between HrS7 and HrS8. (B) Interactions between HrS7 and HrS5. In both figures, the *y*-axis is viral titers (log10), while the *x*-axis shows the HrS7 genotype (A/A= CC003 homozygous A/B is heterozygous, B/B is CC053 homozygous). Within each *x*-axis class the genotypes of HrS8 (*A*) or HrS5 (*B*) are binned left to right (CC003/CC003; CC003/CC053; CC053/CC053).

Given the number of loci segregating within this cross, we next assessed if there was evidence of epistatic interactions between these loci. We found strong support for additive interactions between *Hrs5* and *Hrs6* for day 3 weight loss (LOD = 8.27, genome-wide *P* = 0.05 threshold = 6.25), and also between *Hrs5* and *Hrs9* for hemorrhage (LOD = 9.43, genome-wide *P* = 0.05 threshold = 6.4). We found evidence for a full model of interaction (that is both additive and epistatic interactions) for viral titers between *Hrs7* and *Hrs8* (LOD = 13.3, genome-wide *P* = 0.05 threshold = 11.4), as well as between *Hrs7* and *Hrs5* (LOD = 17.5, genome-wide *P* = 0.05 threshold = 11.4) (Figure 4).

**Figure 5:**
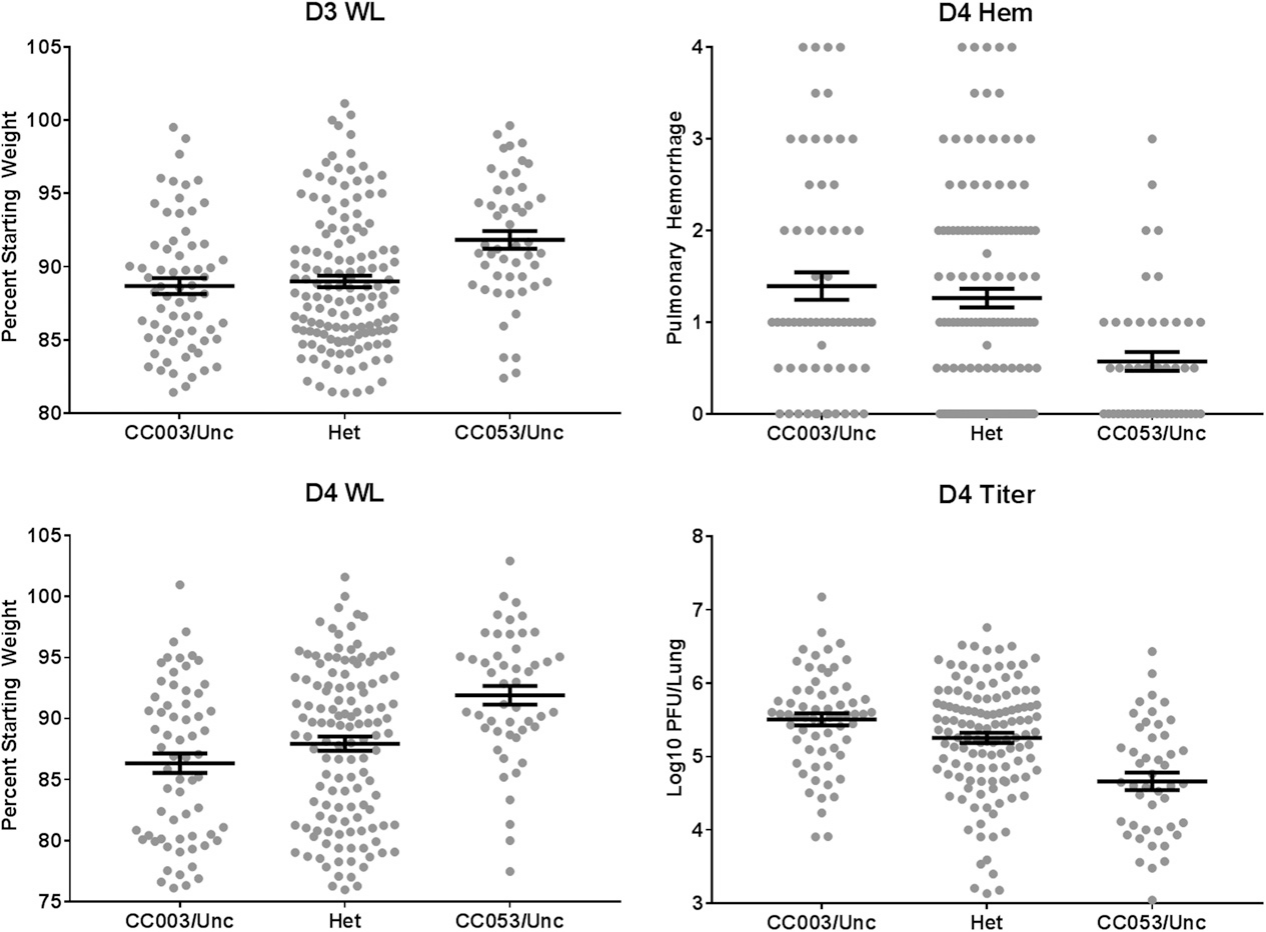
Allele effects. Phenotypes were broken out based on a homozygous CC053/Unc genotype, a homozygous CC003/Unc genotype, or a heterozygous genotype for day 3 and day 4 weight loss, log_10_ viral titer, and hemorrhage at the chromosome 18 QTL.

We determined which ancestral (CC founder strain) alleles were segregating at each QTL in order to better understand the architecture of the QTL and SARS-CoV-associated responses we identified. Throughout the *Hrs5* region, CC003/Unc has a PWK-derived allele, whereas CC053/Unc had a PWK (27.1–31.2 Mb), and then a C57BL/6J (31.2–51) or a C57BL/6J and 129s1/SvImJ (51–58.6 Mb) region of uncertainty (Figure S3). The shared PWK/PhJ haplotype at the proximal end of the locus functionally reduced the QTL region to 31.2–58.6 Mb, and, at the most highly associated marker (JAX00083358, Chr18:51.41 Mb), we found that the CC003/Unc PWK-derived allele was associated with enhanced disease relative to the CC053/Unc C57BL/6J-derived allele—a case of transgressive segregation (Figure 5). *Hrs6* had the CC003/Unc haplotype (C57BL/6J) associated with reduced weight loss as compared to the CC053/Unc haplotype (WSB/EiJ). *Hrs7* showed the CC003/UNC haplotype (PWK/PhJ 55.1–69 Mb; C57BL/6J 69–78 Mb; 129s1SvImJ 78–90 Mb; C57BL/6J and 129s1/SvImJ 90–117.22 Mb) was associated with reduced viral titers as compared to the CC053/Unc haplotype (C57BL/6J and WSB/EiJ uncertainty 55.1–58 Mb; WSB/EiJ and PWK/PhJ uncertainty 58–81 Mb; WSB/EiJ 81–117.22 Mb). *Hrs8* had the CC003/Unc haplotype (NOD/ShiLtJ) associated with lower viral titers than the CC053/UNC haplotype (WSB/EiJ 81.6–88.9 Mb; WSB/EiJ and CAST/EiJ uncertainty 88.9–108 Mb). Lastly, *Hrs9* had the CC003/Unc haplotype (PWK/PhJ centromere–30 Mb, NZO/Hilt and PWK/PhJ uncertainty 30–36 Mb, PWK/PhJ 36–64.4 Mb) associated with lower pulmonary hemorrhage as compared to the CC053/Unc haplotype (NOD/ShiLtJ centromere–22.3 Mb; 129s1/SvImJ 22.3–32.2 Mb; CAST/EiJ 32.2–64.4 Mb).

### Ticam2 plays a critical role in SARS-CoV pathogenesis

**Figure 6:**
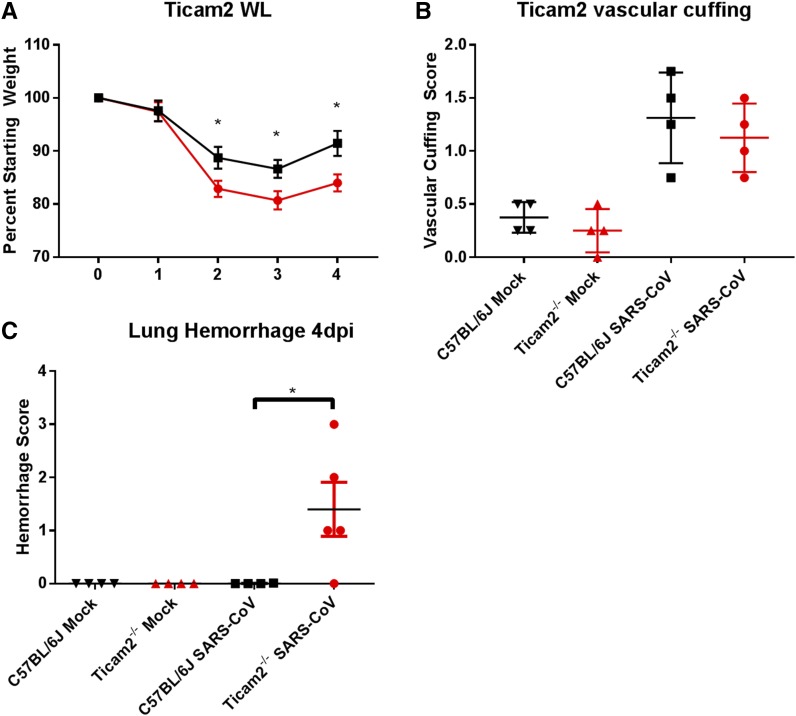
*Ticam2* knockouts. *Ticam2*^−/−^ (*n* = 14) and C57BL/6J (*n* = 12) mice were infected with 10^5^ PFU of MA15 for 4 d. Weight loss data (A) confirms our previously published results (Totura *et al.* 2015). Vascular cuffing in the lung was scored in a blinded manner (B) and pulmonary hemorrhage was scored at the time of tissue harvest (C). Asterisks indicate a *P* value < 0.05.

Given these haplotypic differences, we compared the PWK and C57BL/6J genomes on chromosome *18* from 31.2–58.6 Mb, looking for missense mutations or insertions/deletions to narrow potential candidate genes beneath the QTL. While it is possible that a spontaneous mutation in either CC003/Unc or CC053/Unc is the cause of *Hrs5*, we considered this to be the less likely scenario, and focused our initial bioinformatics analysis based on genotyping of the CC founder lines. Using the publically available Sanger sequences (Oreper *et al.* 2017), we identified 743 missense mutations but no insertions or deletions in the consensus region encompassing 158 coding genes (Table S3). Further examination revealed that four of the missense mutations were located within *Ticam2*, formerly known as *TRAM*—a TLR adapter protein. Previous work by our group identified critical roles for the TLR pathways and adaptors in modulating SARS-CoV disease (Sheahan *et al.* 2008; Totura *et al.* 2015), and led us to further pursue the role of *Ticam2* in contributing to SARS-CoV pathogenesis. Although knockout mice do not test the effect of allelic variation in candidate genes, they can confirm the overall importance of a given gene in phenotypes of interest. In this case, *Ticam2*-deficient mice (*Ticam2*^−/−^) had greater SARS-CoV induced weight loss than C57BL/6J control mice (Figure 6A and raw data in Table S2) [and as previously described (Totura *et al.* 2015)]. While *Ticam2*^−/−^ mice had similar virus titers to C57BL/6J control mice at day 4 postinfection [(Totura *et al.* 2015) and further confirmed in data not shown], their virus load is significantly higher at day 2 postinfection (Totura *et al.* 2015). We further examined *Ticam2*^−/−^ mice for the additional phenotypes that mapped to the same region of chromosome *18*. Initially focusing on vascular cuffing, *Ticam2*^−/−^ mice showed no notable increase in scoring relative to wild-type control mice despite their increased weight loss (Figure 6B). In contrast, pulmonary hemorrhage scores were significantly higher in *Ticam2*^−/−^ mice at 4 d postinfection (Figure 6C). Overall, these data demonstrate an important role for *Ticam2* in SARS-CoV pathogenesis, although additional loci contribute to variation in disease severity.

## Discussion

Genetic reference panels, especially those MPPs with multiple founder strains, have increasingly been seen as a rich tool for understanding mammalian disease states and biomedically important traits in the context of naturally occurring genetic variation (Nedelko *et al.* 2012; Rasmussen *et al.* 2014; Rogala *et al.* 2014). In contrast to previous genetic mapping studies using the entire CC population (Ferris *et al.* 2013; Gralinski *et al.* 2015), we focused on two strains exhibiting extreme responses to SARS-CoV infection, predicting that they would have multiple QTL driving these extreme responses. Further, we hypothesized that each of those QTL would contrast alleles from unique pairs of founder haplotypes (as these susceptibility responses are outside the range of responses seen in other inbred strains). Our study found five QTL impacting SARS-CoV disease responses in this cross. Although each locus in an F2 cross can only contrast two haplotypes; across these five loci were components of seven of the eight CC founder strains, with most contrasting alleles being between classical laboratory haplotypes *vs.* one of the wild-derived inbred founder haplotypes. Our approach highlights the utility in combining novel genetic reference populations with classical F2 crosses in order to more fully probe the complex genetic architecture of disease responses.

Our results also demonstrate the importance of *Ticam2* in control of multiple aspects of SARS-CoV pathogenesis such as weight loss, viral titer, and pulmonary hemorrhage (Figure 6, (Totura *et al.* 2015)). While we did not observe any changes in vascular cuffing in *Ticam2*^−/−^ mice relative to C57BL/6J controls, this is likely due to the complexity of confirming functional allelic variation in a knockout mouse model. Ticam2—a TLR sorting adapter protein—recruits the signaling adapter protein TRIF to mediate TLR4 signaling (Fitzgerald *et al.* 2003; Oshiumi *et al.* 2003). While the ligand that activates TLR4 signaling following SARS-CoV infection has not yet been identified, our group has recently shown that TLR4 deficient mice are highly susceptible to SARS-CoV infection (Totura *et al.* 2015). Other laboratories have demonstrated the importance of TLRs in the host immune response to porcine epidemic diarrhea virus (PEDV) (Cao *et al.* 2015) and mouse hepatitis virus (Mazaleuskaya *et al.* 2012), supporting a general requirement for TLR signaling in an effective immune response to coronavirus infection. TLR4 is classically known as the LPS receptor (Poltorak *et al.* 1998), but it can also recognize host proteins that have altered expression under conditions of cell stress, such as heat shock proteins and proteins involved in the extracellular matrix (ECM) like fibrinogen, heparin sulfate, and hyaluronic acid (Okamura *et al.* 2001; Smiley *et al.* 2001; Cohen-Sfady *et al.* 2005; Akbarshahi *et al.* 2011). Additionally, Wang and Liu recently demonstrated that the SARS-CoV membrane protein stimulates interferon induction in a TRAF3-independent manner, using an as yet unknown TLR (Wang and Liu 2016). We have previously shown that extensive ECM remodeling occurs following SARS-CoV infection (Gralinski *et al.* 2013), and we speculate that either ECM changes, or sensing of the SARS-CoV membrane protein, are likely drivers of TLR4 activation.

Mutations in various TLRs and adapter proteins can have significant impacts on immunity and susceptibility to infectious diseases. *Ticam2* is required for vesicular stomatitis virus induced TLR4-dependent signaling (Georgel *et al.* 2007). Three single nucleotide polymorphisms (SNPs) in *Ticam2* were recently shown to be associated with Tuberculosis susceptibility, and one SNP was associated with resistance (Hall *et al.* 2015). While nonsynonymous mutations in TLRs are rare in human populations [for example, most mutations in the extracellular domain of TLR4 are found in <1% of population (Smirnova *et al.* 2001)], when observed, they can have a profound effect on the host response to infection. For example, the relatively common Asp299Gly mutation in TLR4 has been shown to interfere with recruitment of MyD88 and TRIF to TLR4, and thus diminish downstream NF-kB- and IRF3-mediated signaling (Figueroa *et al.* 2012); individuals with this mutation are more prone to septic shock (Lorenz *et al.* 2002) as well as Crohn’s disease and ulcerative colitis (Cheng *et al.* 2015). It was recently shown that the F protein of respiratory syncytial virus (RSV) binds to, and activates, TLR4 (Rallabhandi *et al.* 2012), and increased RSV disease severity is associated with the Asp299Gly TLR haplotype (Caballero *et al.* 2015).

Ticam2 facilitates the binding of TLR4 and TRIF through the interaction of the TIR domains of the three proteins (Enokizono *et al.* 2013). There are four missense mutations between the C57BL/6J and PWK *Ticam2* sequences; however, because the mutations all occur before the TIR domain, they are unlikely to interfere with Ticam2 binding to either TLR4 or TRIF. The amino-terminal domain of Ticam2 is less well studied, but is known to contain both myristoylation and phosphorylation sites that are essential for Ticam2 to locate to the plasma and endosomal membranes (Rowe *et al.* 2006; Kagan *et al.* 2008). The four *Ticam2* missense mutations between PWK and C57BL/6J are predicted to cause two changes in charge, remove a serine residue, and change a cysteine residue to a serine (S39 is conserved in mammalian species). Those mutations may alter the structure of the Ticam2 amino-terminal domain, impact its membrane localization function, or modify the ability of Ticam2 to properly shuttle TLR4 to the endosome following activation (Kagan *et al.* 2008). Finally, the *Ticam2* locus is genetically complex, encoding overlapping negative regulators that could be impacted by these mutations (Doyle *et al.* 2012). Thus, there are multiple mechanisms by which allelic differences in *Ticam2* could result in functional consequences in TLR4-mediated signaling and immunity. Importantly, while our knockout mouse data confirms the role of *Ticam2* in helping to control SARS-CoV mediated disease, it does not prove that allelic variation in *Ticam2* is the cause of the *HrS5* phenotypes. Continued work is needed to address the possible role of other candidate genes in the *HrS5* interval, and to assess what, if any, functional changes exist between the C57BL/6J and PWK *Ticam2* alleles.

In conclusion, we utilized two strains of the CC showing extreme SARS-CoV responses to identify five host genetic loci driving different aspects of these disease responses. By their design, each CC strain contains haplotype blocks coming from evolutionarily diverse *Mus musculus* substrains. The random sorting of haplotypes that do not share evolutionary history can give rise to extreme phenotypic responses within a strain (Rasmussen *et al.* 2014; Rogala *et al.* 2014; Graham *et al.* 2016) when interacting members of pathways are forced to work with evolutionarily distinct partners. Diverse subspecific alleles were present across the five loci within each of the CC lines, strongly suggesting that the extreme SARS-CoV responses we based our F2 cross on are due, in part, to interactions between alleles from diverse sets of CC founders. Reinforcing the idea that there might be many strain-specific interactions that can drive much of the observed variation in GRPs, in this cross we found evidence for epistatic relationships across three loci in controlling viral load (Figure 4B).

Importantly, we identified a QTL that contributes to multiple SARS-CoV phenotypes, and whole genome sequence analysis pointed to altered function of the innate-immune modulatory gene *Ticam2* as a strong candidate. *Ticam2*^−/−^ mice were used to confirm the role of that gene in contributing to SARS-CoV-induced weight loss and pulmonary hemorrhage, although not vascular cuffing. Knockout mice cannot address the issue of allelic variation, and thus there is a possibility that *Ticam2*^−/−^ mice phenocopy *Hrs5*, and another gene or genes are responsible for the SARS-CoV phenotypes that map to chromosome 18. Use of CRISPR/Cas9 genome editing approaches to swap alleles, rather than ablate genes, and directly testing specific causal mutations in extreme CC strains will be a more relevant way to confirm genetic function in the future. Regardless, this data, along with previously published work (Sheahan *et al.* 2008; Totura *et al.* 2015), combines to demonstrate that TLR recognition of SARS-CoV infection is a crucial part of the host immune response to infection. Because allelic variation in *Tlr4* in humans is frequently associated with increased disease susceptibility, modulating its signaling through use of agonists or antagonists could allow for effective treatment of a number of disease states. Testing drug efficacy in a genetically variable population, including variation in the pathways of interest, is not possible using conventional knockout mice. The genetic variation present in the CC, particularly when it is known to impact functional outcomes such as SARS-CoV susceptibility, would be a particularly rigorous and effective test of proposed human therapeutics that modulate TLR signaling.

## Supplementary Material

Supplemental material is available online at http://www.g3journal.org/content/7/6/1653.supplemental.
