# Supplementary figures and images for: Allelic Variation in the Toll-Like Receptor Adaptor Protein Ticam2 Contributes to SARS-Coronavirus Pathogenesis in Mice
Source: G3 (Bethesda). 2017 Jun 5;7(6):1653–63. doi: 10.1534/g3.117.041434 (PMC5473747; doi:10.1534/g3.117.041434)

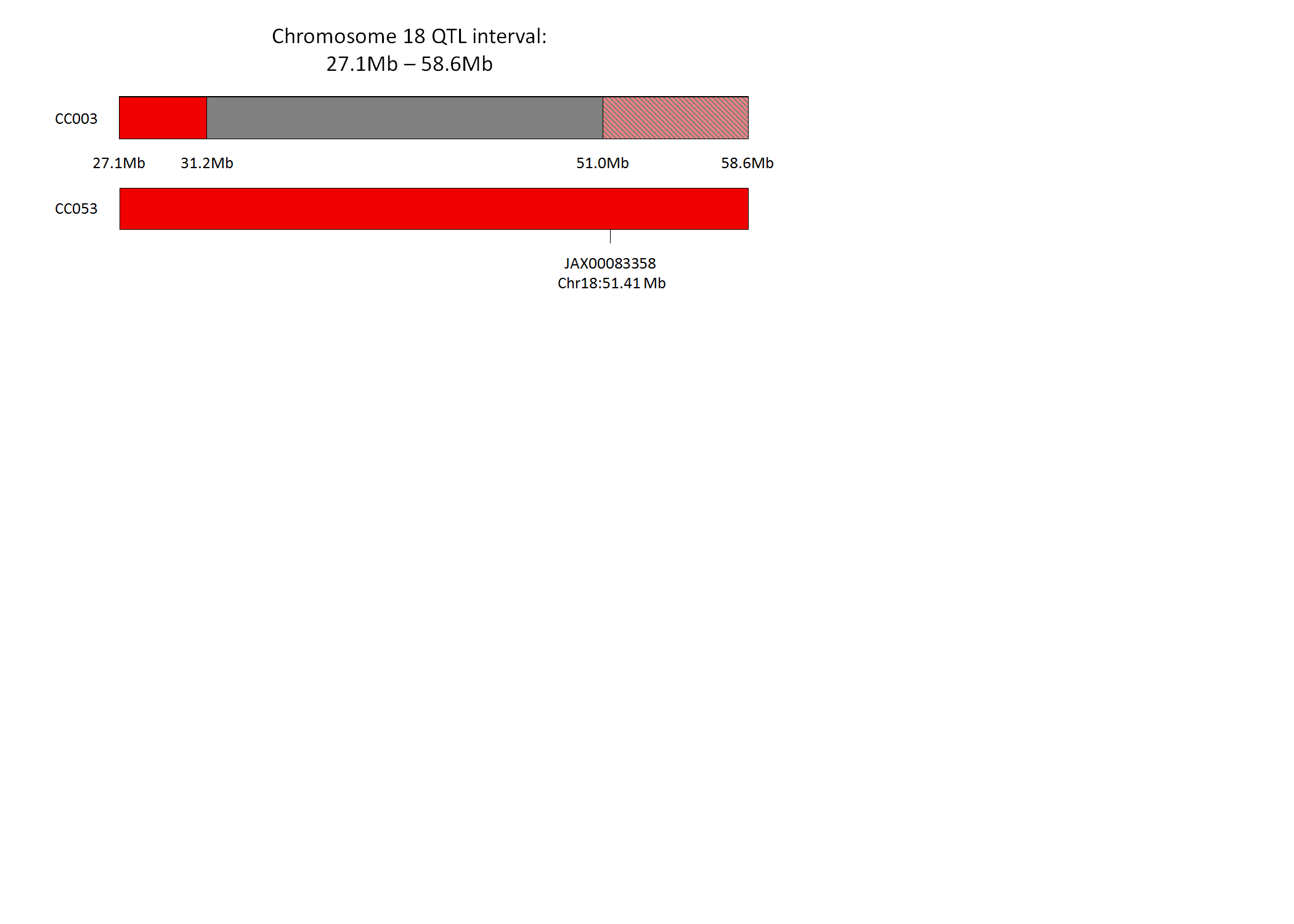

Supplement: Supplementary file 1 [file 1653File001.tif]

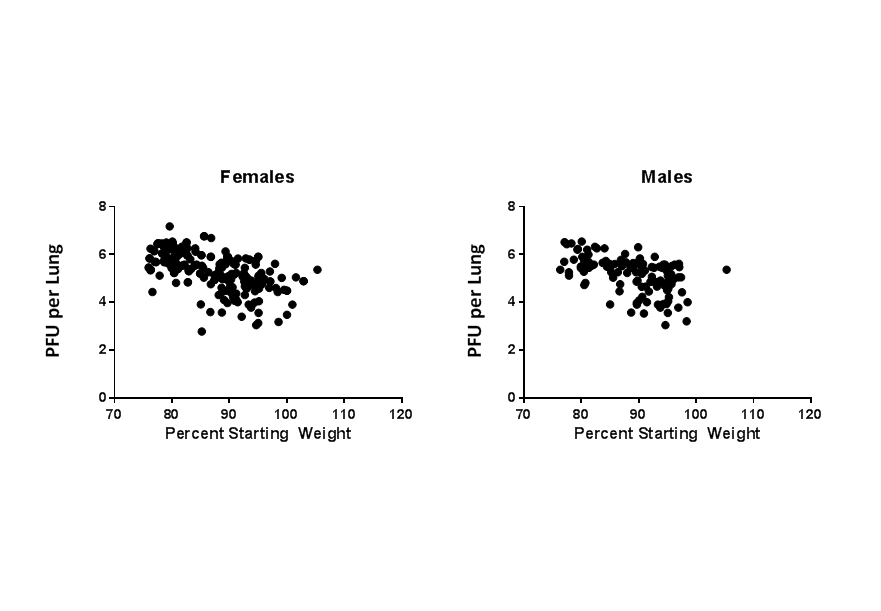

Supplement: Supplementary file 4 [file 1653File004.tif]

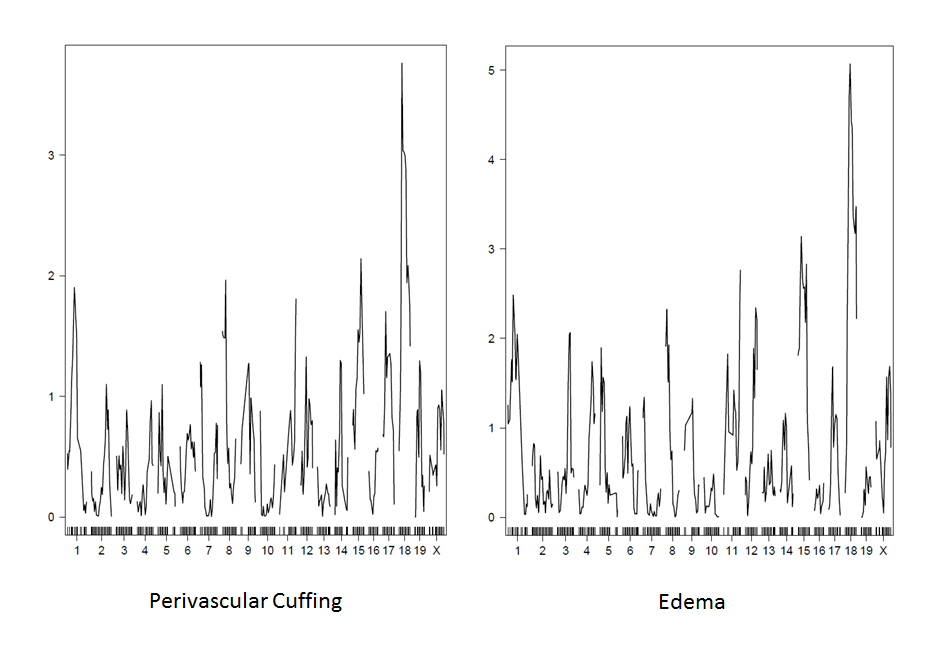

Supplement: Supplementary file 8 [file 1653File008.tif]
